# Reminder Strategies to Improve Meal-Logging Adherence: Protocol for a Microrandomized Trial

**DOI:** 10.2196/100239

**Published:** 2026-10-05

**Authors:** Magdalena Fuchs, Victoria Brügger, Qiuhan Jin, Benjamin Wirth, Stefan Bilz, Florian von Wangenheim, Michael Brändle, Tobias Kowatsch, Mia Jovanova

**Affiliations:** 1 Department of Management, Technology and Economics, ETH Zurich, Weinbergstrasse 56/58, Zürich, Zurich, 8006, Switzerland, +41 446321111; 2 School of Medicine, University of St. Gallen, St. Gallen, Switzerland; 3 Department of Endocrinology, HOCH Health Ostschweiz, St. Gallen, Switzerland; 4 Department of Internal Medicine, HOCH Health Ostschweiz, St. Gallen, Switzerland; 5 Institute for Implementation Science in Health Care, University of Zurich, Zurich, Switzerland

**Keywords:** microrandomized trial, mobile health, push notifications, mHealth, adherence, habit formation, loss aversion, nutrition tracking

## Abstract

**Background:**

Accurate measurement of lifestyle factors is central to understanding how daily behaviors act as risk factors or protective buffers to noncommunicable diseases. While wearable devices enable passive monitoring of physical activity or sleep, nutritional intake still depends on active participant input, such as manual dietary logs or image-based recordings. Adherence to such logging tasks often declines rapidly, impacting data completeness and clinical utility. Theory-based reminders drawing on loss-framing or logging consistency feedback (ie, tracking streaks) can improve adherence to lifestyle data collection, but the effects of these strategies on repeated dietary logging remain unclear.

**Objective:**

The study aims to examine the effects of 2 theory-driven reminder strategies—loss-framing and logging consistency—on adherence to repeated, image-based meal logging.

**Methods:**

We conducted a microrandomized trial embedded within a 4-week observational lifestyle phenotyping study in Switzerland (N=200, age ≥45 y, BMI ≥25 kg/m²). Participants photographed their meals at each mealtime (breakfast, lunch, and dinner) using a mobile app over 28 days. A decision point was scheduled prior to participant-defined habitual mealtimes. Participants were randomly assigned with equal probability to (1) a reminder emphasizing loss of a daily financial reward for not logging (“loss-framing”), (2) a reminder providing feedback on recent logging consistency (“logging consistency”), or (3) a neutral reminder (“active control”). The proximal outcome is whether the participant logs a meal within 2 hours of receiving a reminder. Participants earned a daily financial reward contingent on meal-logging completion. To estimate intervention effects, we will use marginal excursion effect models for binary outcomes, adjusting time-varying covariates (eg, day in study) and baseline covariates (eg, age and gender). Ethics approval for this study was granted by the Cantonal Ethics Committee of Eastern Switzerland (BASEC ID 2025‐00972).

**Results:**

Enrollment began in November 2025, and the study was initiated on December 8, 2025. As of August 2026, 130 participants have been recruited, 120 have been enrolled, and 76 participants have completed the study, with an anticipated completion date of January 2027. Data analysis has not yet begun, and study results are expected to be published in Q2 2027.

**Conclusions:**

By clarifying the proximal effects of loss-framed and consistency-based reminders, findings will inform the design of future digital health studies to improve meal-logging adherence in daily life. This work contributes to the development of scalable, theory-driven reminder strategies for enhancing dietary data quality in observational and interventional research.

## Introduction

### Background

The increased prevalence of commercially available wearable sensing devices and smartphones has opened new avenues for longitudinal health and lifestyle data collection [1,2], specifically in digital biomarker research [3,4]. Smartphones, smartwatches, and smart rings enable continuous monitoring of lifestyle factors in daily life, outside of traditional clinical settings, and with minimal participant burden [4]. However, measurements of key lifestyle factors such as mood or dietary intake continue to rely on self-report, commonly collected through app-based logging [3,5].

Critically, adherence (ie, the proportion of participants’ completed vs missed logs) to app-based logging tasks, such as manual meal logging, remains a persistent challenge in digital health studies [3], as low adherence can compromise data quality and undermine the validity of findings [3,6,7]. Moreover, adherence to data collection behaviors, such as meal logging, often declines over time, with many participants logging only sporadically after the first few days of a study [4,8]. For example, in a large-scale study focused on cardiovascular health, participants logged for a mean duration of only 4.1 days [9]. A growing body of digital health research has begun to examine the use of smartphone reminders about logging consistency (ie, mentioning participants’ prior behaviors or streaks) and the use of financial incentives (ie, monetary compensation for log completion) to improve adherence [4,7,8,10]. However, most adherence-promoting interventions, to date, have failed to ground messaging strategies in health psychology, communication, or behavior change theory [11], making it difficult to identify the potential mechanisms driving observed effects. Moreover, existing studies often evaluate average between-person effects [9,12], leaving open questions about how reminders influence adherence within individuals over time.

The widespread use of consistency reminders and financial incentives targets two known barriers to adherence in digital health data collection and digital meal logging in particular: (1) a lack of sustained motivation over time and (2) a mismatch between desired app-based behaviors (ie, repeated logging) and participants’ daily routines [13]. Two theory-informed mechanisms have been used in the digital health context to address these challenges: loss-framed reminders [12,14,15] and logging consistency reminders [16,17]. Each targets a distinct motivational pathway that may influence adherence to repeated logging behaviors.

Loss-framed reminders are messages that emphasize incentives that participants stand to lose if they do not adhere to the desired behavior (ie, meal logging). This strategy leverages adherence-contingent financial incentives, which have shown considerable success in increasing adherence in prior digital health studies [4,7,8,10]. Such messages have been found to be more effective than gain-framed messages in digital health contexts, such as promoting physical activity [12] and improving medication adherence [18,19]. This differential effectiveness draws on research in behavioral economics showing that individuals are generally more motivated to avoid losses than to pursue equivalent gains, a phenomenon known as loss aversion [20,21]. Prior work suggests that loss-aversion framing can be effective even with relatively small financial stakes [20]. For example, in one comparable study, a loss-framed incentive of US $1.40 per day was more effective in promoting adherence to a daily step-count goal than an equivalently valued gain-framed incentive [12]. By highlighting the forfeiture of a previously allocated incentive if the desired behavior is not performed, loss-framed reminders increase the perceived cost of nonadherence and strengthen the intention to act [18,22]. In behavioral theory, loss-framing primarily targets *reflective motivation* [23], which refers to deliberate, conscious drivers of behavior, including a participant’s intentions and their perceived importance of an action. This motivational pathway is particularly relevant in observational studies, where the behavior serves data collection goals rather than participants’ own health improvement. In these contexts, participants may not see inherent value in the logging behavior, which can make it harder to sustain motivation.

In contrast, logging consistency reminders emphasize participants’ recent logging behavior by providing feedback on their personal logging consistency. Such feedback has been applied in digital health interventions to encourage continued engagement [16,17], and empirical findings suggest that consistency-based feedback can improve adherence by increasing participants’ awareness of their progress and reinforcing the target behavior (meal logging) [24]. By drawing attention to recent performance (ie, percentage of meals logged), this approach aims to stabilize routines by increasing perceived regularity and strengthening associations between contextual cues (mealtimes) and the desired behavior (logging meals) [14,17,22,25], which is a central mechanism to habit formation [17]. While the 28-day study period is likely too short to form long-term habits [26], automaticity develops incrementally rather than at a fixed threshold [26-28], so these cue-behavior mechanisms may begin to operate on a shorter timescale. In behavioral theory, this process supports *automatic motivation*, which refers to habitual processes that trigger behavior in response to contextual cues without requiring sustained conscious effort [23]. Automatic motivation is particularly relevant in digital data collection contexts, where the target behavior is not yet embedded in users’ daily routines [13] and reflective motivation alone may be insufficient, as participants may not be consciously thinking about intentions or incentives at the precise moment logging is needed (ie, right before or during a meal). Together, loss-framed reminders and logging consistency reminders therefore target complementary motivational pathways—reflective and automatic motivation, respectively—representing 2 theoretically distinct strategies for supporting meal-logging adherence.

Despite widespread use of adherence reminder interventions in digital health studies, key constructs such as loss aversion and logging consistency feedback have not been explicitly applied to improve adherence in digital health data collection. While both financial incentives and consistency feedback have strong grounding in theory and empirical evidence, many digital health studies use such features [4,7,8,10] without subjecting them to rigorous empirical testing. In particular, loss-framing, despite being a powerful and established tool for influencing behavior, has not yet been systematically evaluated as a means of motivating participants to maintain adherence to data collection protocols in observational research. Similarly, while logging consistency feedback is sometimes used in digital health studies, its specific role in reinforcing routine logging behaviors remains untested. Overall, there is limited evidence on the proximal outcomes of message framing on data collection protocol adherence, especially in comparison to neutral or generic message alternatives. Understanding the proximal effects of such strategies is, however, critical, as adherence behaviors fluctuate daily and are shaped by momentary states and contexts [29]. These time-varying patterns need to be captured to identify which intervention options are most effective, for whom, and under what conditions. Currently, this knowledge remains limited in digital health research.

This study addresses the gaps by examining whether theory-informed messaging interventions improve proximal adherence to a meal-logging observational study protocol. We focus on 2 proposed psychological mechanisms: loss framing, which draws on behavioral economics to enhance reflective motivation, and logging consistency feedback, designed to support automatic motivation through routine formation. To our knowledge, this is the first study to explicitly test theory-driven reminder framing in the context of image-based dietary logging.

### Objectives and Research Questions

The study addresses the following research questions (RQs): (1) What is the proximal effect of delivering a loss-framed reminder, compared to a neutral reminder, on adherence to a meal-logging protocol in 2 hours following each randomized decision point? (2) What is the proximal effect of delivering a logging consistency reminder, compared to a neutral reminder, on adherence to a meal-logging protocol in 2 hours following each randomized decision point? and (3) Does the proximal effect on logging adherence significantly differ between loss-framed reminders and logging consistency reminders?

We hypothesize that (H1) loss-framed reminders increase logging adherence relative to neutral reminders and (H2) logging consistency reminders increase logging adherence relative to neutral reminders. For RQ3, we test whether the effects of the 2 reminder types differ without specifying a directional hypothesis. Secondary objectives include heterogeneity analyses examining whether reminder effectiveness changes over the study period and whether within-person physiological patterns (eg, heart rate variation [HRV]) moderate response rates to reminders, with the aim of identifying temporal and physiological markers of receptivity to behavioral prompts.

## Methods

### Study Procedures

#### Study Setting

The study is conducted at the School of Medicine at the University of St. Gallen in collaboration with HOCH Health Ostschweiz, St. Gallen, Switzerland. This microrandomized trial (MRT) is embedded within the parent *Glow Up* project [30], a larger observational study aiming to test the feasibility of digital biomarkers for prediabetes screening and to develop digital subphenotypes of diabetes risk, using wearable devices and continuous glucose monitors, which is beyond the scope of the current study. The current MRT specifically examines adherence to the meal-logging component of the observational protocol, which requires participants to take a photo of each of their 3 daily meals over 28 days. Recruitment is ongoing, with participant enrollment running from November 2025 to November 2026 (expected). This protocol is reported in accordance with the SPIRIT (Standard Protocol Items: Recommendations for Interventional Trials) 2013 guidelines [29] (Checklist 1).

#### MRT Design

An MRT is an experimental design originally developed to inform the construction of just-in-time adaptive interventions [31,32]. Unlike a traditional randomized controlled trial, in which each participant is randomized once to a single condition, an MRT randomizes intervention options repeatedly at predefined decision points. In this study, the 3 theory-driven reminder types, loss-framed, logging consistency, and active control (a neutral reminder referencing neither incentives nor recent logging behavior), are randomized independently for each participant at each scheduled mealtime (decision point). Consequently, each participant may receive all 3 reminder types across the study.

This repeated within-person randomization allows for estimation of the causal effect of each reminder type on the proximal outcome, defined as whether the participant logs a meal in the 2 hours following the reminder [31,32]. It also permits the examination of whether reminder effectiveness changes over the study period, indicating possible habituation or fatigue effects [31,33]. Additionally, the collection of wearable data during the intervention period allows us to explore whether within-person physiological patterns moderate the effectiveness of each reminder type. This contextual evidence forms the empirical groundwork for future adaptive systems that tailor reminder delivery to the individual [32,34]. Table 1 summarizes the key MRT design concepts and their operationalization in this study.

**Table 1.** Key terms of a microrandomized trial (MRT) and their operationalizations in the present study.

Key term	Operationalization
Distal outcome	High degree of data completeness (average of >75% of meals logged) throughout the remote intervention period (28 d)
Proximal outcome	Completion (yes/no) of a meal log in the 2 hours following a reminder message
State of vulnerability	An individual is considered vulnerable if a meal has not been logged by the time a notification is scheduled within their specified mealtime window
Decision points	Three times daily over the 28-day period (84 decision points in total) at breakfast, lunch, and dinner, within the first 30 minutes of a participant’s predefined mealtime window; the participant is randomly assigned to one of the 3 intervention options
Intervention options	Three options: *Loss-framed* reminder message, *logging consistency* reminder message, and *neutral* reminder message (active control)
Tailoring variables	All intervention options: participants’ typical mealtimes (indicated as 2-h intervals). Option *logging consistency* only: participants’ logging consistency up to the current decision point (ie, percentage of meal logs that have been completed since they enrolled in the study out of a total of 3/d)
Decision rules	A message is delivered if the participant is in a state of vulnerability (ie, has not logged by the time a notification is scheduled within their specified mealtime window)

#### Participants and Recruitment

Consistent with the parent *Glow Up* project, individuals are considered eligible to participate if they are (1) Swiss citizens or permanent residents, (2) at least 45 years old, (3) have a BMI of 25 or higher, (4) do not have a mental health condition that prevents them from independently giving informed consent, (5) can read German and own or have reliable access to a smartphone with a data plan, and (6) are capable of using smartphone apps. Exclusion criteria include current or a history of stroke, heart disease, renal failure, cancer, or diabetes (type 1 or 2); past vascular bypass surgery or angioplasty; current or planned use of glucose-lowering medications; pregnancy or breastfeeding; skin conditions at wearable placement sites; and unwillingness or inability to wear a smartwatch and smart ring for the full 28-day study period, including during sleep and work, following the larger, parent *Glow Up* project [30]. Participants are recruited through information material distributed via community boards, post offices, supermarket blackboards, flyers, in-store promotions, social media, and company or institutional networks (eg, intranets or newsletters). Recruitment activities are maintained throughout the enrollment period until the target sample size is reached, with recruitment materials directing interested individuals to an online eligibility questionnaire. As part of this questionnaire, participants self-report age and sex and provide their contact information (email and phone number). Eligible respondents are contacted by the study team to confirm interest and schedule an in-person onboarding visit.

#### Study Procedure

The MRT study protocol consists of 3 main phases. First, participants complete an on-site onboarding visit followed by a 4-week remote MRT and data collection period, and a final, on-site follow-up visit. The timeline of recruitment and study procedures is summarized in Figure 1.

During the onboarding visit, participants install and register with the *MyDataHelps* [35] app (CareEvolution, LLC), a mobile app for clinical studies, which is used to complete all study tasks, including questionnaires and image-based meal logging. Participants are first instructed to complete an in-app questionnaire reporting their typical breakfast, lunch, and dinner times at 2-hour intervals, for weekdays and weekends separately. These data are then used to adapt the timing of reminder messages in the MRT. Specific instructions and meal-logging interfaces can be seen in Multimedia Appendix 1.

During the remote intervention period, participants complete 3 daily meal logs during predefined meal windows (customized during the onboarding visit) for 28 days, though logs can also be submitted outside these windows to account for additional meals or snacks. Each image-based meal-logging event is expected to take no longer than 30 seconds. Upon initiating a meal log in the app, participants are prompted to capture an image before eating (Figure S1 in Multimedia Appendix 1), though they can also provide additional information in a free-text format. Breakfast and lunch logs collect no additional data, while dinner logs include 2 follow-up questions assessing overall mood and perceived stress using single-item 10-point Likert-type scales (1-10; Figure S3 in Multimedia Appendix 1). If a participant skips a meal, they are asked to indicate a “skipped meal” within the app, triggering a 3-question survey on reasons for skipping (Figure S2 in Multimedia Appendix 1). These follow-up questions aim to ensure equivalent response effort across conditions, thereby discouraging systematic use of the meal skip option. Adherence is measured as the number of completed meal logs, including skipped-meal reports. As part of the parent project protocol, participant lifestyle data (sleep, physical activity, blood glucose, etc) are tracked using wearable devices (Ultrahuman Ring AIR and Ametris Leap) throughout the entire 28-day remote intervention period.

Following the MRT, participants are invited for a final on-site visit, where they complete an in-app follow-up questionnaire assessing perceived helpfulness and disruptiveness of reminders, satisfaction with reminder timing and frequency, subjective preference for message framing, and perceived burden of the data collection protocol and wearable device (Multimedia Appendix 1). Additional procedures conducted during the onboarding and follow-up visits, including blood draws and wearable device setup and retrieval, are part of the parent study and described in detail in the parent study protocol [30].

**Figure 1.**
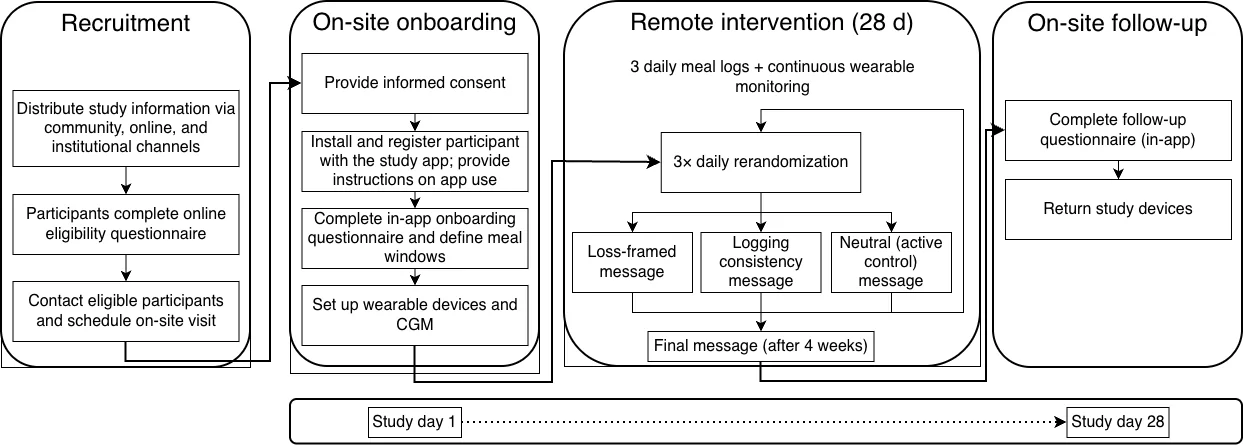
Participant flow and schedule of study procedures. Participants undergo eligibility screening before continuing on to on-site onboarding, which starts the 28-day remote intervention period, during which they complete meal logging and wearable-based data collection and receive randomized reminder messages up to 3 times daily. The study concludes with an on-site follow-up visit and questionnaire assessing reminder acceptability and data collection burden. CGM: continuous glucose monitor.

Participants receive CHF 50 for completing both in-person visits (CHF 25 each) and can earn up to an additional CHF 100 based on meal logging adherence (1 CHF=approximately US $1.21 as of September 2026). The adherence payment is calculated as CHF 100 multiplied by the proportion of the 28 study days on which at least 2 meal logs are completed. Thus, each nonadherent day reduces the adherence payment by CHF 3.57, rounded to CHF 3.60 in the final payment. For example, a participant who completes at least 2 meal logs on 23 of 28 days (82% adherence) and attends both in-person visits receives CHF 132 in total, out of a maximum possible CHF 150. This stake is comparable in size to previous studies, demonstrating the effectiveness of loss aversion with financial incentives (US $1.40 [12] and US $2 [36] lost for each day of nonadherence to a behavioral goal).

Pilot experience (14 d, 10 participants) prior to the main study indicated that these procedures are feasible to implement, and responses to the follow-up questionnaire on disruptiveness of reminders and perceived burden of the data collection protocol identified no major concerns regarding participant acceptability.

### MRT Design

#### Decision Points, Randomization, and Proximal Outcome

Each mealtime (breakfast, lunch, and dinner) over the 28-day period serves as an individual decision point in the MRT, resulting in up to 84 decision points in total per participant at which reminder messages may be delivered automatically through the *MyDataHelps* study app. The MRT workflow is first triggered randomly within the first 30 minutes of the participant-specified 2-hour meal window. This randomization allows for some daily variation, helping to prevent habituation effects, while still ensuring that the reminder intervention occurs early in the mealtime window. If the participant has already logged the respective meal, they are no longer in a vulnerable state (Table 1), and no reminder is sent to avoid message fatigue [37]. Otherwise, the participant is randomized with equal probability (1/3 each) into one of 3 intervention options: *loss-framed*, with reminders focusing on the loss of the daily financial incentive in case of noncompletion of the meal logs; *logging consistency,* with reminders providing logging consistency feedback based on their previous logging behavior; or *neutral (active control*), with reminders mentioning the need to log a meal and omitting cues to financial incentives or meal-logging consistency. At each decision point, the intervention option is assigned independently using NumPy’s random sampling function (*numpy.random.choice*) in a back-end function, with equal allocation probabilities of 1/3 to each condition. Randomization is memoryless and independent of assignments at all prior decision points, with no adjustment based on participant history, engagement, or covariates. Participants are not informed of the specific intervention condition assigned at each decision point, although they are aware that they may receive different reminder types. Reminder messages are automatically delivered to participants through the *MyDataHelps* study app, and no study personnel are involved in intervention assignment or message delivery at individual decision points.

A positive proximal outcome (1) is defined as meal log completion within 2 hours of receiving a reminder message (Table 1). Noncompletion of the meal log or completion outside this window is considered a negative outcome (0), as they cannot be reliably attributed to the reminder. In the absence of a gold standard for defining proximal outcome time windows, different MRTs rely on domain-specific considerations to determine appropriate timeframes. Reported windows vary widely from 30 minutes to over 24 hours [31,32,38,39]. Given that individual meal timing can fluctuate by approximately 1 hour across months and up to 3 hours from day to day, as measured over 3 months [40], we define a 2-hour window following reminder delivery as the key proximal outcome interval, that is, to examine immediate reminder effects on meal logging. Overall, this window aims to balance flexibility (natural meal timing fluctuations) and temporal proximity to the intervention (short-term reminder effects). A flowchart of the MRT design logic is shown in Figure 2.

**Figure 2.**
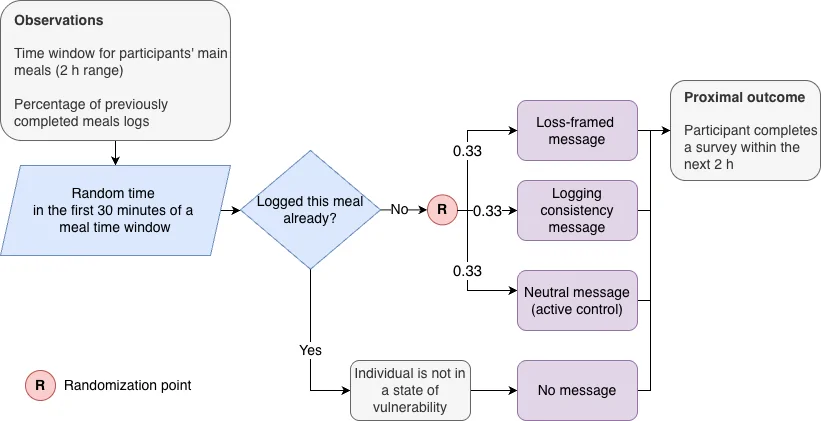
Flowchart of the microrandomized trial (MRT) timing and randomization algorithm, messaging intervention, and proximal outcomes.

Through the data collection platform (*MyDataHelps*) and wearable devices, the following data are collected for analysis: meal type (breakfast, lunch, and dinner); timestamp and type of notification (not available if no notification is sent); timestamp of meal log completion; wearable sensors: heart rate, HRV, step count, active minutes, calories burned, sleep duration, and sleep stages. Notification assignment and delivery information and meal-log completion timestamps are automatically recorded using the *MyDataHelps* platform, while wearable-derived measures are collected continuously using the Ultrahuman Ring AIR and Ametris Leap devices. Meal type, notification time and type, and meal-log completion will be used to answer the primary RQs 1 to 3 (proximal effects of loss-framed, logging consistency, and neutral reminders on meal-log completion), while data collected from wearable devices will be used to investigate secondary objectives (examining whether within-person physiological patterns moderate response rates to reminders).

By randomizing message content at each decision point, the MRT design allows us to estimate the proximal effects of specific messaging strategies on adherence within individuals over time, including whether these effects diminish due to habituation.

By randomizing message timing, the MRT enables secondary analyses of state-dependent treatment effects, including whether physiological signals such as HRV at the time of delivery moderate reminder effectiveness, providing insights into when participants may be more vs less receptive to behavioral prompts.

#### MRT Intervention Options

##### Message Development

For each intervention option—*neutral* (active control), *loss-framed*, and *logging consistency* messages—a bank of 15 distinct reminder message texts was developed to capture the theoretical construct of interest beyond sampling variability and to minimize message fatigue due to repetitive messaging. Message content was initially generated with support from a large language model (OpenAI GPT-4o) and refined by the research team to ensure alignment with the intended theoretical constructs. Importantly, we pretested the message banks through manipulation checks with 100 German-speaking online participants recruited via Prolific (Prolific Academic Ltd) [41]. Following the literature [42], participants were asked to rate each message across two items: (1) “This statement emphasizes the loss of a reward when a meal is not logged*”* and (2) “This statement emphasizes building a routine when logging meals,” using a 7-point Likert scale (1=“strongly disagree,” 7=“strongly agree”). A message was retained if the intended construct was rated an average of >5 on a 7-point Likert scale, and nontarget constructs were rated <4. Similarly, *neutral* (active control) were only retained if both constructs were rated <4 on average. This procedure confirmed that the message variants successfully operationalize their intended construct. Final German message banks (alongside English translations) for each intervention option are provided in Table S1 in Multimedia Appendix 1. At each MRT decision point, following random assignment to an intervention option, one reminder is randomly selected from the corresponding 15-message bank using NumPy’s random sampling function (*numpy.random.choice*) and delivered via the *MyDataHelps* app.

##### Loss-Framing

We operationalize loss aversion through a small daily financial incentive (CHF 3.6 as a reward that is forfeited if meal logging is not completed. This approach is intended to increase the salience of meal logging under conditions of low intrinsic motivation [18,22]. All participants (regardless of assigned intervention option) receive a daily financial incentive of 3.6 CHF contingent upon completing at least 2 image-based meal logs that day. The loss-framed intervention messages employ framing that exclusively emphasizes the potential forfeiture of this daily financial reward if meal logging is not adhered to. These reminders are delivered according to the MRT logic (Figure 2) at predetermined decision points and highlight the risk of losing the immediate financial incentive without referencing prior logging consistency. Example messages demonstrating this singular focus on incentive loss are presented in Textbox 1. The standardized call-to-action (*“Log your meal now!"*) is maintained across all messages to match other intervention options.

Textbox 1. **Example messages for th*e “loss-framed”* message group translated to English (original language: German).**

*Missing today means losing out on your payout*. *Log your meal now!*

*Your daily cash reward disappears if you don’t act. Log your meal now!*

*Don’t forget to log your meal today – or you’ll miss out on your daily bonus. Log your meal now!*

*No photo means no bonus – secure your points now. Log your meal now!*

*Don’t forget to log your meal today – or you’ll miss out on your daily bonus. Log your meal now!*

##### Logging Consistency

Logging consistency reminders are tailored based on the participant’s recent logging adherence rate. For highly adherent participants (≥75% logging rate), messages emphasize the potential disruption to their established meal-logging routine. For less consistently adherent participants (<75% logging rate), messages emphasize the potential to regain consistency by logging their food. This framing is designed to increase perceived behavioral regularity and strengthen cue-action associations around mealtimes, which can increase the likelihood of repetition, particularly when it draws attention to personal progress or disruptions in the behavioral pattern [14,17,22,25]. For highly adherent participants (≥75% logging rate), messages emphasize the potential disruption to their established meal-logging routine. For less consistently adherent participants (<75% logging rate), messages emphasize the potential to regain consistency by logging their food. This framing is designed to increase perceived behavioral regularity and strengthen cue-action associations around mealtimes. The call-to-action (“Log your meal now!”) remains consistent across all intervention options, and there is no reference to financial incentives. Table 2 displays example messages for the “logging consistency” intervention option.

**Table 2.** Example messages for consistent (≥75% of logs) and inconsistent (<75%) logging users in the *“*logging consistency” message group translated to English (original language: German).

Previous consistency	Logging consistency reminder messages
≥75%	*You’ve logged 87% of your meals so far—don’t break your flow. Log your meal now!* *76% of your meals are documented already. Skipping today would break your routine. Log your meal now!* *You’ve been doing great (90% logged), but skipping today means losing your streak. Log your meal now!* *82% of your meals logged so far—stay on track. Log your meal now!* *With a tracking rate of 79%, it would be a shame to break the streak today. Log your meal now!*
<75%	*You’ve only logged 50% of meals. Today is your chance to rebuild your streak! Log your meal now!* *You’re at just 47% tracking—start building your habit today. Log your meal now!* *Your tracking rate is only 63% but logging today gets you back on track. Log your meal now!* *Only 47% of meals logged so far—it takes a bit more for a real routine to form. Log your meal now!* *Your current tracking rate is 47%—time to rebuild your rhythm. Log your meal now!*

##### Active Control

To isolate the specific effects of loss-framed and logging consistency messaging, an active control option consisting of neutral reminders is implemented. These reminders are used to test whether meal-logging adherence is influenced by the actual framing of messages (*“*loss-framed” or “logging consistency”) rather than by the mere presence of a reminder. Control messages make no reference to participants’ past behavior or potential rewards/losses. The call to action is kept identical across all intervention options to ensure that any observed differences in adherence can be attributed to message framing rather than to variations in urgency or directive strength. Examples of neutral reminder messages are shown in Textbox 2.

Textbox 2. **Example messages for neutral reminders translated to English (original language: German).**

*Remember to input your food intake today. Log your meal now!*

*Don’t forget to monitor what you eat today. Log your meal now!*

*Time to track your food. Log your meal now!*

*Your nutrition diary is waiting. Log your meal now!*

*Have you eaten today? Log your meal now!*

##### Supplementary Reminders

To maintain continuous data collection, participants receive two types of reminder messages outside of the MRT messaging structure: (1) automated prompts every 48 hours reminding participants to check battery levels on their wearable devices and charge them if needed and (2) a troubleshooting message in case no data transmission is detected from their wearable device for more than 6 hours. This minimizes the number of messages received while proactively preventing gaps in the data collection.

### Measures

#### Proximal Outcome

The primary proximal outcome is a time-varying binary indicator of meal-logging adherence. At each decision point, the outcome is coded as 1 if the participant completes the corresponding meal log within 2 hours after receiving the randomized reminder message and 0 if the participant does not complete the meal log.

#### Time-Varying Covariates

Measured time-varying covariates include the day of the study, the type of meal, and the cumulative number of reminders received.

#### Time-Fixed Covariates

We measure the time-fixed covariates age and gender.

### Data Management

Meal logs and reminders (delivery, timestamps, and type of reminder) are electronically collected through the *MyDataHelps* platform. Wearable data are collected via platforms of the wearable device providers (Ultrahuman, Ametris) and linked using study-specific participant identifiers. Directly identifying information is stored separately from research data and access is restricted to authorized study personnel in accordance with the procedures of the parent *Glow Up* study. Further details on storage, security, pseudonymization, and access procedures are provided in the parent study protocol [30].

### Data Analysis

#### Effects of Loss-Framed and Logging Consistency Reminders Compared to Neutral Reminders

To address the RQs, we will use MRT-appropriate methods with time-varying interventions and binary proximal outcomes. The analyses aim to estimate the effects of the 2 different reminder strategies on self-monitoring adherence, accounting for repeated measures, within-subject correlation, and time-varying availability. Our analytic approach closely follows that of Bell et al [39], applying marginal excursion effect (MEE) estimation in the context of an MRT with a binary proximal outcome [32].

To evaluate the proximal effect of loss-framed reminders (RQ1) and the effect of logging consistency feedback (RQ2) compared to active control reminders, the MEE for MRTs with binary outcomes will be estimated, as described in Qian et al [32] and Bell et al [39]. The proximal outcome is a binary, time-varying measure of adherence to the meal-logging protocol, defined as the completion of a meal log within 2 hours of receiving a randomized reminder. The MEE quantifies the effect of receiving an active intervention reminder message (“loss-framed” or “logging consistency*”*) vs a neutral (active control) message at a given time point, assuming participants followed the randomization schedule up to that time. The effect will be estimated on the log relative risk scale and marginalized over all decision points and participants [39].

The primary model will adjust for day in study (coded numerically from 1 to 28), age (continuous), and gender (categorical). Exploratory analyses will examine effect modification by day in study, meal type (breakfast, lunch, and dinner; categorical), age, and gender using moderated causal excursion effect models. *P*<.05 will be considered statistically significant.

#### Differential Effect Between the Active Intervention Conditions

To assess whether the 2 active intervention options differ in their effect on logging adherence (RQ3), the loss-framed intervention option will be compared to the logging consistency option using the same MEE estimator. This analysis tests for a differential proximal effect between 2 theoretically distinct mechanisms: reflective motivation (loss framing) and automatic motivation (logging consistency feedback).

As with RQ1 and RQ2, the proximal outcome is the completion (yes/no) of a meal log within 2 hours of the reminder, and the effect will be estimated on the log relative risk scale and averaged across all eligible decision points. The model will include the same covariate specification and exploratory analyses as the RQ1 and RQ2 models.

#### Sample Size Considerations

Sample size calculations were performed using the sample size formula for MRTs with binary outcomes [43]. The study design includes 28 days of participation with 3 decision points per day and a randomization probability of one-third per decision point. We assumed a constant expected availability of 0.7 (ie, participants are expected to be vulnerable to receive notifications at 70% of decision points), following sample size considerations in the foundational *HeartSteps* MRT [34,44], which assumed 50% to 70% availability and subsequently observed approximately 80% availability in the collected trial data. The success probability without intervention was modeled to decline log-linearly from 0.7 at the start to 0.4 at the end of the study, reflecting a drop in spontaneous adherence over time. A starting adherence rate of 0.7 is conservative relative to initial adherence rates of 70% to 93% reported in comparable diet and lifestyle self-monitoring studies [45,46]. Adherence in these studies declined over time, dropping to approximately 50% to 60% within the first month [46]. We therefore assumed a decline from 0.7 to 0.4 over the 28-day study period, toward the lower end of observed adherence trajectories, to ensure that we estimate a sample size sufficient to detect the hypothesized effect. The proximal treatment effect was assumed to decrease log-linearly from a relative risk of 1.1 to 1.0, reflecting a gradual reduction in intervention effectiveness over the course of the study, for example, due to habituation effects and reduced novelty. Given the uncertainty around the magnitude of the proximal treatment effect in this setting, we deliberately specified a conservative effect trajectory to ensure that the study would be adequately powered to detect even relatively small effects. This assumption is cautious relative to prior MRT power calculations; for example, the *Drink Less* MRT [47] considered average proximal relative risks of 1.50 to 2.16 for opening the app within 1 hour of a notification, with effects declining over the 30-day study period. All assumptions used in the sample size calculation, including availability, baseline success probabilities, and the proximal treatment effect trajectory, were specified before examining trial outcome data.

With a significance level of 0.05 and a desired power of 90%, the trial requires a sample size of 159 participants to have sufficient sensitivity to detect the hypothesized proximal effects on meal log completion. Each of the 3 pairwise comparisons (RQ1-RQ3) was specified a priori as a distinct scientific question and will be evaluated separately at a 2-sided significance level of .05. Accordingly, results will be reported and interpreted marginally for each comparison rather than as a joint confirmatory claim. Therefore, we do not apply a multiplicity adjustment across the 3 comparisons [48]. Since the trial aims to recruit a sample of 200 participants, the MRT is adequately powered under our assumptions, which model both baseline adherence and intervention effects as declining over the study period.

#### Interim Analysis

An interim analysis will be conducted after approximately 20% of the planned sample (N=40) has completed the trial. The study authors will perform this analysis to examine patterns in adherence, feasibility, and data completeness. Interim results will be disseminated through conferences, consistent with the dissemination of final study findings.

### Technical Contingencies

In case of technical problems with sending/receiving reminders (eg, due to low connectivity of the participant’s device or a failed message delivery via *MyDataHelps*), failures are handled according to the stage at which they occur. Availability is assessed before randomization at each decision point according to whether the corresponding meal has already been logged. If the participant is unavailable, no randomization is performed. This prerandomization availability indicator is incorporated into the MEE estimator used for the primary analysis and is also reflected in the sample-size calculation, which assumes availability at 70% of decision points. If randomization occurs but message delivery subsequently fails, the assigned condition and delivery status remain recorded, and the decision point is not retrospectively reclassified as unavailable.

In the rare event of a technical problem with the randomization back end (eg, a server-side failure preventing execution), this will be identifiable in the data as an absence of expected notification records; affected decision points will be excluded from analysis. Randomization at subsequent decision points proceeds independently of previous assignments or technical failures.

### Ethical Considerations

This study was reviewed and approved by the Cantonal Ethics Committee of St. Gallen (approval was granted in June 2025). Prior to participation, all individuals provide written informed consent, including consent for participation, collection of wearable and app-based data, and data storage and sharing in accordance with the General Data Protection Regulation and Swiss Data Protection Law. Participants are informed of their right to withdraw at any time without penalty. Participants are not informed about the specific intervention variants delivered at each decision point to avoid influencing behavior; however, they are aware that they may receive different types of reminders during the study. No data monitoring committee was established, as the study involves a low-risk behavioral intervention. Study data will be made available upon reasonable request and subject to appropriate data use agreements. Important protocol modifications will be submitted to the ethics committee and updated in the trial registration where applicable.

### Trial Registration

The study includes a microrandomized component embedded within a larger observational study. Data collection for the parent observational study began on December 8, 2025, and the parent study was registered on ClinicalTrials.gov on January 19, 2026 (NCT07373418); prospective trial registration was not initially considered a requirement for the observational parent study, and no outcome data had been examined at the time of registration. The microrandomized component was not initially recognized as requiring separate registration from the parent study and was retrospectively registered on April 21, 2026 (NCT07555262), once this requirement was identified. Before registration of the microrandomized component, no treatment-effect or outcome-by-condition analyses had been conducted, and randomized intervention assignments had not been examined in conjunction with outcome data. The primary outcome definition, randomization parameters, proximal outcome window, and primary analysis model were prespecified before data collection, with the corresponding analysis code committed to a public, version-controlled repository on September 23, 2025 [49]. The repository contains only pilot data used to develop and test the analysis code; no outcome data from the main study were available at the time. These prespecified elements were not subsequently changed in response to outcome data.

## Results

Funding for the study was approved in October 2025. Enrollment began in November 2025, and the study was initiated on December 8, 2025. As of August 2026, 130 participants have been recruited, 120 have been enrolled, and 76 participants have completed the study procedures. The study is anticipated to be completed in January 2027. Data analysis has not yet begun, and study results are expected to be published in Q2 2027.

## Discussion

### Overview

By systematically testing the effects of loss-framed and logging consistency reminders on adherence to image-based meal logging, this study can provide new theory-informed evidence on how messaging strategies influence proximal adherence to an observational data collection study in digital health. Critically, the MRT design allows for direct, head-to-head comparison of 2 theoretically distinct but commonly applied adherence strategies within the same trial, an approach that remains rare in the digital health literature, where these strategies are typically evaluated in isolation or applied without systematic comparison [4,7,8,10]. Furthermore, to our knowledge, this is the first trial to evaluate theory-based reminder-framing strategies for adherence to image-based dietary logging specifically. Unlike retrospective self-report methods, image-based logging requires real-time capture (ie, users must remember to photograph meals at the moment of eating, as missed meals cannot be recorded after consumption). Given this distinct adherence demand [50], this trial addresses an underexplored gap in the dietary assessment literature.

From a theory perspective, this is the first study to isolate the proximal effects of loss-framed incentive reminders and consistency-based behavioral feedback on lifestyle data collection behavior in daily life. While existing work has applied smartphone reminders referencing logging consistency [16,17] or financial incentives [7,8,10] to improve adherence, these components have not been experimentally disentangled. Our findings are expected to provide empirical evidence on how distinct motivational pathways—loss aversion versus routine reinforcement—drive short-term engagement in image-based meal-logging tasks.

Practically, findings can inform the design of future digital health protocols by identifying reminder strategies that are both effective and feasible to implement at scale. Most digital health studies rely on high participant adherence to ensure data quality yet offer little guidance on how to maintain logging behavior over time. Our results will provide empirical evidence on whether and what kind of reminder messages based on different behavioral framings can improve adherence to a repeated behavior, image-based meal logging. This insight can further support the development of evidence-based reminder interventions that can be tested across study designs and different behaviors (eg, symptom reporting).

### Limitations

Several limitations to this study should be acknowledged. First, the relatively short study duration (28 d) limits our ability to assess the sustainability of observed effects over longer periods. The formation of new routines and long-term changes in behavior often unfold over longer periods of around 10 weeks [27,28], and it remains to be investigated whether the proximal effects of reminder framing observed in this study translate into durable routines beyond this intervention time. Future studies should aim to replicate these findings in extended data collection periods. Second, the primary outcome (adherence to meal logging) is measured as a binary task-completion variable indicating whether a participant logged a meal within 2 hours after receiving a reminder. While this allows for an objective measure of adherence, it does not capture more nuanced engagement, such as how many minutes after receiving a reminder participants respond to them by completing the logging behavior and how this response time may change throughout the day (ie, for different mealtimes). Future work could further integrate passive indicators (eg, app interaction patterns) or self-report measures (eg, perceived burden and motivation type) to characterize engagement patterns. Third, this study is conducted in a research study context, where participants receive financial compensation tied to their adherence. This may limit the generalizability of the findings to settings where incentives are absent or less salient. Further research is needed to assess whether the effects of reminder framing persist in contexts where incentives are provided less frequently (eg, not daily) or take nonfinancial forms. Fourth, this study relies on image-based meal logging, where participants record meals by taking photos rather than manually entering food items, as in previous trials [51]. This may lower the effort required compared to traditional text-based logging. As a result, findings may not be directly comparable to studies using text-based logging.

### Conclusions

This MRT evaluates and compares the effects of 2 theoretically grounded reminder strategies, loss-framing and logging consistency feedback, on participant adherence to digital meal logging. Findings from this trial have the potential to improve data completeness in digital health studies, which are increasingly used in observational and interventional clinical research on nutrition, metabolic health, and chronic disease prevention. By testing mechanisms that target reflective and automatic motivation, the study will provide evidence on how to design low-burden, scalable adherence interventions that support more reliable self-monitoring in clinical settings. These insights are directly relevant to researchers and clinicians seeking to improve data quality and completeness in digital health studies and remote patient monitoring protocols.

## Supplementary material

10.2196/100239 Multimedia Appendix 1 App interfaces, meal-logging workflow, questionnaires, and reminder message bank.

10.2196/100239 Checklist 1 SPIRIT checklist.

## Abbreviations

HRV: heart rate variation
MEE: marginal excursion effect
MRT: microrandomized trial
RQ: research question
SPIRIT: Standard Protocol Items: Recommendations for Interventional Trials
