# Supplementary material for: Reminder Strategies to Improve Meal-Logging Adherence: Protocol for a Microrandomized Trial
Source: JMIR Res Protoc. 2026 Oct 5;15:e100239. doi: 10.2196/100239 (PMC13637989; doi:10.2196/100239)
Supplement: Supplementary file 1 [file resprot-v15-e100239-s001.docx]

## Multimedia Appendix 1

**Figure S1** Screenshot of the in-app image-based meal logging interface (original in German) with English translations. If the participant selects that they have skipped this meal, they continue directly to the skipped-meal survey.

**Figure S2** Screenshot of the in-app questions in case a meal is skipped. A value on the Likert scales can be selected by clicking on the desired number on the scale.

**Figure S3** Additional questions for participants when they complete the dinner meal log.

**Table S1** Final message bank. Manipulation checks were conducted on the original German versions of all messages. English translations are provided here for reporting purposes only. The placeholder [adherence score] represents a participant-specific value computed dynamically at the time of message delivery, defined as the proportion of days since enrollment on which the participant recorded at least two meal logs.

| **Condition** | **Message ID** | **Content (German)** | **Content (English translation)** |
| --- | --- | --- | --- |
| **loss-framed** | loss_00 | Ohne Mahlzeitendokumentation heute verlierst du deinen Bonus. Trage jetzt deine Mahlzeit ein! | Without meal documentation today, you'll lose your bonus. Log your meal now! |
|  | loss_01 | Ohne Foto keine Auszahlung – du verlierst deinen Tagesbonus. Trage jetzt deine Mahlzeit ein! | No photo, no payout – you'll lose your daily bonus. Log your meal now! |
|  | loss_02 | Kein Foto, kein Geld – sichere dir deinen Bonus. Trage jetzt deine Mahlzeit ein! | No photo, no money – secure your bonus. Log your meal now! |
|  | loss_03 | Nur wer heute einträgt, bekommt die Belohnung, sonst verlierst du sie. Trage jetzt deine Mahlzeit ein! | Only those who log today get the reward, otherwise you'll lose it. Log your meal now! |
|  | loss_04 | Wer heute nichts einträgt, verzichtet auf den Tagesbonus. Trage jetzt deine Mahlzeit ein! | Anyone who doesn't log today misses out on the daily bonus. Log your meal now! |
|  | loss_05 | Wenn du heute nichts einträgst, ist dein Bonus weg. Trage jetzt deine Mahlzeit ein! | If you don't log anything today, your bonus is gone. Log your meal now! |
|  | loss_06 | Lass kein Geld liegen – dokumentiere jetzt dein Essen. Trage jetzt deine Mahlzeit ein! | Don't leave money on the table – document your meal now. Log your meal now! |
|  | loss_07 | Kein Eintrag heute bedeutet kein Geld für dich. Trage jetzt deine Mahlzeit ein! | No entry today means no money for you. Log your meal now! |
|  | loss_08 | Verliere nicht den heutigen Bonus. Trage jetzt deine Mahlzeit ein! | Don't lose today's bonus. Log your meal now! |
|  | loss_09 | Du lässt Geld liegen. Wenn du heute nicht diene Mahlzeiten dokumentierst, ist der Bonus weg. Trage jetzt deine Mahlzeit ein! | You're leaving money behind. If you don't document your meals today, the bonus is gone. Log your meal now! |
|  | loss_10 | Der Bonus von heute geht verloren, wenn du nichts trackst. Trage jetzt deine Mahlzeit ein! | Today's bonus will be lost if you don't track anything. Log your meal now! |
|  | loss_11 | Verlier' deinen heutigen Bonus nicht – denk an den Eintrag. Trage jetzt deine Mahlzeit ein! | Don't lose your daily bonus – remember to log your entry. Log your meal now! |
|  | loss_12 | Keine Mahlzeit dokumentiert = keine Tagesbelohnung. Trage jetzt deine Mahlzeit ein! | No meal documented = no daily reward. Log your meal now! |
|  | loss_13 | Heute nichts einzutragen bedeutet auf den Bonus zu verzichten. Trage jetzt deine Mahlzeit ein! | Not logging anything today means missing out on the bonus. Log your meal now! |
|  | loss_14 | Kein Eintrag, kein Geld – der Tagesbonus verfällt sonst. Trage jetzt deine Mahlzeit ein! | No entry, no money – the daily bonus will expire otherwise. Log your meal now! |
| **consistency feedback - low adherence (<75%)** | context_low_00 | Nur [adherence score] % deiner Mahlzeiten sind bisher dokumentiert – du kannst jederzeit wieder in den Rhythmus finden. Trage jetzt deine Mahlzeit ein! | Only [adherence score] % of your meals have been documented so far – you can get back into the rhythm at any time. Log your meal now! |
|  | context_low_01 | Deine Trackingrate liegt nur bei [adherence score] % – ein Eintrag heute hilft dir, wieder in eine Routine zu finden. Trage jetzt deine Mahlzeit ein! | Your tracking rate is only [adherence score] % – logging today will help you get back into a routine. Log your meal now! |
|  | context_low_02 | Bisher hast du [adherence score] % deiner Mahlzeiten eingetragen. Ein Eintrag heute ist ein Schritt zurück zur Regelmäßigkeit. Trage jetzt deine Mahlzeit ein! | You've logged [adherence score] % of your meals so far. One entry today is a step back toward consistency. Log your meal now! |
|  | context_low_03 | Es sind bisher [adherence score] % dokumentiert – heute ist ein guter Zeitpunkt, um wieder Regelmäßigkeit aufzubauen. Trage jetzt deine Mahlzeit ein! | [adherence score] % have been documented so far – today is a good time to rebuild regularity. Log your meal now! |
|  | context_low_04 | Du bist bei [adherence score] % – bring' deine Routine wieder ins Laufen. Trage jetzt deine Mahlzeit ein! | You're at [adherence score] % – get your routine back on track. Log your meal now! |
|  | context_low_05 | Bisher sind [adherence score] % getrackt. Heute ist der perfekte Moment, um den Faden wieder aufzunehmen. Trage jetzt deine Mahlzeit ein! | [adherence score] % tracked so far. Today is the perfect moment to pick up where you left off. Log your meal now! |
|  | context_low_06 | [adherence score] % getrackt – das ist zu wenig für eine stabile Routine. Trage jetzt deine Mahlzeit ein! | [adherence score] % tracked – that's not enough for a stable routine. Log your meal now! |
|  | context_low_07 | Mit nur [adherence score] % Trackingrate bisher ist es schwer, eine stabile Routine zu entwickeln. Trage jetzt deine Mahlzeit ein! | With a tracking rate of only [adherence score] % so far, it's hard to build a stable routine. Log your meal now! |
|  | context_low_08 | Du hast bislang [adherence score] % der möglichen Mahlzeiten dokumentiert – mit einem Eintrag heute findest du zurück in die Routine. Trage jetzt deine Mahlzeit ein! | You've documented [adherence score] % of possible meals so far – one entry today will help you get back into the routine. Log your meal now! |
|  | context_low_09 | Du hast bisher nur [adherence score] % getrackt – regelmäßige Einträge helfen dir dein Trackingverhalten zu stabilisieren. Trage jetzt deine Mahlzeit ein! | You've only tracked [adherence score] % so far – regular entries help you stabilize your tracking behavior. Log your meal now! |
|  | context_low_10 | Bisher sind nur [adherence score] % deiner Mahlzeiten getrackt – nutze die heutige Gelegenheit, deine Routine zurückzugewinnen. Trage jetzt deine Mahlzeit ein! | Only [adherence score] % of your meals have been tracked so far – use today's opportunity to reclaim your routine. Log your meal now! |
|  | context_low_11 | [adherence score] % deiner Mahlzeiten sind bisher getrackt – mit einem Eintrag heute stärkst du deine Routine. Trage jetzt deine Mahlzeit ein! | [adherence score] % of your meals have been tracked so far – one entry today strengthens your routine. Log your meal now! |
|  | context_low_12 | Bei bisher [adherence score] % geloggten Mahlzeiten fehlt dir noch die Regelmäßigkeit. Fang' heute wieder an. Trage jetzt deine Mahlzeit ein! | With [adherence score] % of meals logged so far, you're still missing consistency. Start again today. Log your meal now! |
|  | context_low_13 | [adherence score] % hast du bisher getrackt. Das reicht noch nicht für eine feste Gewohnheit. Trage jetzt deine Mahlzeit ein! | You've tracked [adherence score] % so far. That's not yet enough to build a solid habit. Log your meal now! |
|  | context_low_14 | Deine Trackingrate liegt momentan bei [adherence score] % – Zeit, wieder Rhythmus aufzubauen. Trage jetzt deine Mahlzeit ein! | Your tracking rate is currently at [adherence score] % – time to rebuild your rhythm. Log your meal now! |
| **consistency feedback - high adherence (≥75%)** | context_high_00 | Deine Tracking-Serie steht bei [adherence score] %. Unterbrich sie jetzt nicht. Trage jetzt deine Mahlzeit ein! | Your tracking streak stands at [adherence score] %. Don't break it now. Log your meal now! |
|  | context_high_01 | Du hast dir mit einer Trackingrate von [adherence score] % eine echte Routine aufgebaut – brich sie nicht ausgerechnet heute. Trage jetzt deine Mahlzeit ein! | You've built a real routine with a tracking rate of [adherence score] % – don't break it today of all days. Log your meal now! |
|  | context_high_02 | Aktuell hast du [adherence score] % getrackt – unterbrich deinen Flow nicht. Trage jetzt deine Mahlzeit ein! | You're currently at [adherence score] % tracked – don't interrupt your flow. Log your meal now! |
|  | context_high_03 | Du hast [adherence score] % deiner Mahlzeiten bisher dokumentiert – das ist eine starke Serie, lass sie nicht reißen! Trage jetzt deine Mahlzeit ein! | You've documented [adherence score] % of your meals so far – that's a strong streak, don't let it slip! Log your meal now! |
|  | context_high_04 | Du bist konstant dabei – unterbrich deinen Lauf mit [adherence score] % Trackingrate nicht heute. Trage jetzt deine Mahlzeit ein! | You've been consistently on it – don't break your run of [adherence score] % tracking rate today. Log your meal now! |
|  | context_high_05 | Unterbrich nicht deine Trackingserie: Du bist bei [adherence score] %. Trage jetzt deine Mahlzeit ein! | Don't break your tracking streak: you're at [adherence score] %. Log your meal now! |
|  | context_high_06 | Du hast eine starke Trackingserie aufgebaut: [adherence score] %. Wenn du heute auslässt, reißt sie. Trage jetzt deine Mahlzeit ein! | You've built a strong tracking streak: [adherence score] %. If you skip today, it breaks. Log your meal now! |
|  | context_high_07 | Du warst bei [adherence score] % der Mahlzeiten dabei – stark! Beende nicht deine Serie. Trage jetzt deine Mahlzeit ein! | You've been there for [adherence score] % of meals – impressive! Don't end your streak. Log your meal now! |
|  | context_high_08 | Du warst bisher in [adherence score] % der Mahlzeiten konsequent – heute auszusetzen bricht deine Statistik. Trage jetzt deine Mahlzeit ein! | You've been consistent in [adherence score] % of meals so far – skipping today breaks your record. Log your meal now! |
|  | context_high_09 | Eine Lücke heute und deine starke Routine von [adherence score] % Tracking verliert an Stabilität. Trage jetzt deine Mahlzeit ein! | One gap today and your strong routine of [adherence score] % tracking loses its momentum. Log your meal now! |
|  | context_high_10 | Du hast bereits [adherence score] % dokumentiert – brich deine starke Serie nicht ab. Trage jetzt deine Mahlzeit ein! | You've already documented [adherence score] % – don't break your strong streak. Log your meal now! |
|  | context_high_11 | Du hast aktuell eine [adherence score] % Trackingquote. Lass das nicht heute scheitern, sonst verlierst du deinen guten Lauf. Trage jetzt deine Mahlzeit ein! | You currently have a [adherence score] % tracking rate. Don't let it fall apart today, or you'll lose your great run. Log your meal now! |
|  | context_high_12 | Du hast bisher [adherence score] % deiner Mahlzeiten eingetragen – heute auszusetzen würde deine Serie brechen. Trage jetzt deine Mahlzeit ein! | You've logged [adherence score] % of your meals so far – skipping today would break your streak. Log your meal now! |
|  | context_high_13 | Du hast bereits [adherence score] % deiner Mahlzeiten dokumentiert. Beende deine Serie nicht jetzt. Trage jetzt deine Mahlzeit ein! | You've already documented [adherence score] % of your meals. Don't end your streak now. Log your meal now! |
|  | context_high_14 | Du hast dir mit [adherence score] % eine super Routine aufgebaut – verspiel sie nicht heute. Trage jetzt deine Mahlzeit ein! | You've built a great routine with [adherence score] % – don't throw it away today. Log your meal now! |
| **neutral (active control)** | control_00 | Denk kurz an dein Ernährungstagebuch. Trage jetzt deine Mahlzeit ein! | Take a moment to think about your food diary. Log your meal now! |
|  | control_01 | Zeit für deinen Eintrag! Trage jetzt deine Mahlzeit ein! | Time for your entry! Log your meal now! |
|  | control_02 | Hast du heute schon gegessen? Trage jetzt deine Mahlzeit ein! | Have you eaten today yet? Log your meal now! |
|  | control_03 | Noch nichts notiert? Hol’s nach. Trage jetzt deine Mahlzeit ein! | Haven’t logged anything yet? Catch up now. Log your meal now! |
|  | control_04 | Schon gegessen? Dann trag’s jetzt ein. Trage jetzt deine Mahlzeit ein! | Already eaten? Then log it now. Log your meal now! |
|  | control_05 | Vergiss nicht ein Foto deiner Mahlzeit zu machen. Trage jetzt deine Mahlzeit ein! | Don’t forget to take a photo of your meal. Log your meal now! |
|  | control_06 | Wie sieht’s heute mit dem Essen aus? Trage jetzt deine Mahlzeit ein! | How’s it going with meals today? Log your meal now! |
|  | control_07 | Nicht vergessen, dein Essen zu tracken. Trage jetzt deine Mahlzeit ein! | Don’t forget to track your food. Log your meal now! |
|  | control_08 | Denk dran, heute noch dein Essen zu dokumentieren. Trage jetzt deine Mahlzeit ein! | Remember to document your food today. Log your meal now! |
|  | control_09 | Es ist wieder Zeit für einen Eintrag. Trage jetzt deine Mahlzeit ein! | It’s time for another entry. Log your meal now! |
|  | control_10 | Zeit, dein Essen zu dokumentieren. Trage jetzt deine Mahlzeit ein! | Time to document your meal. Log your meal now! |
|  | control_11 | Denk daran, deine Mahlzeit heute einzutragen. Trage jetzt deine Mahlzeit ein! | Remember to log your meal today. Log your meal now! |
|  | control_12 | Ein schneller Check-in heute? Trage jetzt deine Mahlzeit ein! | A quick check-in today? Log your meal now! |
|  | control_13 | Kurze Erinnerung: Vergiss nicht, heute ein Foto deiner Mahlzeit zu machen. Trage jetzt deine Mahlzeit ein! | Quick reminder: don’t forget to take a photo of your meal today. Log your meal now! |
|  | control_14 | Es ist Zeit dein Essen zu dokumentieren. Trage jetzt deine Mahlzeit ein! | It’s time to document your meal. Log your meal now! |

**Follow-up Questionnaire (Section: Adherence and Burden)**

1. How helpful were the reminder notifications in keeping you engaged with the study (completing nutrition logs)?

Likert scale 1 (not helpful at all) – 5 (very helpful)

2. How often did the reminders come at a time that was convenient for you?

Likert scale 1 (never) – 5 (always)

3. How disruptive were the reminders to your daily routine?

Likert scale 1 (not disruptive at all) – 5 (very disruptive)

4. How would you rate the frequency of reminders you received throughout the study?

Likert scale 1(not frequent enough) – 5 (too frequent)

5. What aspect of the reminders made the biggest difference to you (in terms of encouraging you to complete the nutrition surveys)? (Select from a drop-down menu)

- Convenient timing
- Message tone
- Message content/wording
- Reminder frequency
- None / All the same

6. What type of message were you most motivated by to complete your meal logs? (Select from a drop-down menu)

- Loss-framed: Messages that reminded me of the potential loss of the financial compensation.
- Logging consistency: Messages that emphasized building/maintaining a routine around meal logging.
- Neutral: Messages that were impersonal and neutral.
- None / All the same

7. How much burden was added to your day-to-day life through the daily surveys?

Likert scale 1 (no burden at all) – 5 (extremely burdensome)

8. How much burden was added to your day-to-day life through the wearable device?

Likert scale 1 (no burden at all) – 5 (extremely burdensome)

9. How much burden was added to your day-to-day life through the CGM?

Likert scale 1 (no burden at all) – 5 (extremely burdensome)

10. How much burden was added to your day-to-day life through the in-person visits?

Likert scale 1 (no burden at all) – 5 (extremely burdensome)

11. Please provide any other feedback you may have regarding any element of the study (in-person visit, observational period, devices, questionnaires) to tell the study team how to improve in the future:

[Free text answer format]
